# Facility-level integration of hypertension and diabetes services with HIV treatment in sub-Saharan Africa: Observational evidence from Malawi, South Africa, and Zambia

**DOI:** 10.1371/journal.pone.0346029

**Published:** 2026-04-01

**Authors:** Linda Sande, Mariet Benade, Timothy Tchereni, Aniset Kamanga, Vinolia Ntjikelane, Allison Morgan, Taurai Makwalu, Wyness Phiri, Priscilla Lumano-Mulenga, Prudence Haimbe, Hilda Shakwelele, Amy Huber, Sophie Pascoe, Mhairi Maskew, Nancy Scott, Sydney Rosen

**Affiliations:** 1 Health Economics and Epidemiology Research Office, School of Clinical Medicine, Faculty of Health Sciences, University of the Witwatersrand, Johannesburg, South Africa; 2 Department of Global Health, Boston University School of Public Health, Boston, Massachusetts, United States of America; 3 Clinton Health Access Initiative-Malawi, Lilongwe, Malawi; 4 Clinton Health Access Initiative-Zambia, Lusaka, Zambia; 5 Zambia Ministry of Health, Lusaka, Zambia; University of Cape Town Faculty of Science, SOUTH AFRICA

## Abstract

**Background:**

A growing number of people living with HIV (PLHIV) also have non-communicable diseases (NCDs). Shifting to an integrated delivery model may facilitate care-seeking and improve outcomes for people with a dual burden of HIV and NCDs. We describe the current state of integration of hypertension and diabetes care into HIV treatment in Malawi, South Africa and Zambia.

**Methods:**

We administered structured interviews to HIV treatment providers in 41 primary healthcare facilities to evaluate how NCD care is provided to PLHIV accessing antiretroviral therapy (ART). We defined integration as provision of NCD services within the HIV clinic. The potential degree of integration in HIV clinics ranged from not integrated at all (no NCD services) to fully integrated (all NCD services). We also surveyed a sample of ART clients about their access to integrated care.

**Results:**

The degree of integration varied across the facilities and countries. All facilities in South Africa reported being fully integrated for HIV care and hypertension and diabetes, and most providers in South Africa identified no barriers to integration. Integration was much less complete in Malawi and Zambia, with most facilities offering hypertension and diabetes screening/diagnosis and support but limited treatment or disease monitoring services. Frequently cited barriers to integration in Malawi and Zambia were limited staff knowledge of integrated care provision and facility space constraints. ART clients’ experience with integrated services mirrored provider responses. Over 90% of survey participants in South Africa reported HIV and non-HIV visit and medication collection alignment, compared to less than half in Malawi and Zambia.

**Conclusions:**

The level of integration of hypertension and diabetes care with HIV treatment varies widely across facilities in Malawi, South Africa, and Zambia, despite each country having national guidelines that promote integration. Interventions to increase integration must consider differences among facilities at baseline.

## Introduction

An increasing proportion of people living with HIV (PLHIV) require care for other chronic conditions, in addition to antiretroviral therapy (ART) for HIV. Globally, the population living with HIV is growing older and is thus at a higher risk for age-related non-communicable diseases (NCDs), as well as suffering the direct long-term consequences of HIV [1–4]. Among PLHIV, the prevalence of hypertension has been reported to range from 10–27% and the prevalence of diabetes is estimated at 2–5% [5–9]. Sub-Saharan Africa (SSA), where roughly two thirds of the world’s PLHIV reside [10], has increasingly reported a rising burden of both NCDs [6,8].

In many countries in SSA, healthcare systems have traditionally managed noncommunicable conditions separately from HIV [11,12]. Vertical programs for HIV, which primarily arose from global efforts and targeted funding to address the HIV crisis, generally have separate service locations within primary healthcare facilities, different clinic visit schedules, and even different providers dedicated to HIV patients [11–13]. NCD care is usually provided in the general outpatient clinic, which may or may not also offer maternal and child healthcare and/or acute healthcare [14].

While separating HIV treatment from services for other chronic conditions may have facilitated expansion of HIV services historically [15], it also creates a number of disadvantages. In settings in which regular preventative healthcare screening is not the norm, isolating HIV treatment from care for other conditions misses opportunities to diagnose and manage those conditions in comorbid HIV patients. Separating HIV and NCD services is also inefficient for clients and likely for facilities, due to the larger number of single-purpose clinic visits and overlapping services, such as medication dispensing, required [12,16]. Integration of these services, in contrast, is expected to promote the provision of comprehensive and consistent care, increase case-finding, enhance adherence to treatment, optimize retention in care, and make more efficient use of shared resources, such as diagnostic supplies and infrastructure [12,17–22].

Because of the drawbacks of maintaining separate services and potential advantages of combining them, integration of NCD services into HIV care has become a widely accepted goal, recommended by the World Health Organization (WHO) [17,18] and included in many national HIV care guidelines [23–26]. Integration has proven difficult and slow to achieve in practice, however, and progress towards integrated care at the facility level remains uneven despite the existence of national guidelines [27–30]. While many examples of specific models of integration supported by external funders are described in the literature [31], little has been reported about how integrated service delivery functions in routine, non-study settings.

To understand how integration is being operationalized in primary healthcare facilities, we conducted an exploratory survey of healthcare providers and ART patients with comorbidities at primary healthcare facilities in three countries in sub-Saharan Africa: Malawi, South Africa, and Zambia. Our objective was to assess on-the-ground variation in these countries’ levels of integration of hypertension and diabetes into HIV care and obstacles they face in integrating care, as a starting point for considering opportunities for improvement in HIV-NCD integration at the facility level.

## Methods

### Overview

All three countries in this study have national guidelines for integration of HIV and NCD care at public sector healthcare facilities. Guidelines in Malawi and Zambia are similar, with ART clinics within facilities asked to offer NCD services to HIV patients. In Malawi, 2022 guidelines on the “Clinical management of HIV in children and adults” call for HIV-NCD integration during group health information talks offered before consultations, screening for and management of NCDs as part of HIV treatment, combined storage of NCD and HIV files, and alignment of clinic visits for HIV and NCD treatment [32]. Similarly, the “2020 Zambia consolidated guidelines for treatment and prevention of HIV infection” also recommend screening for hypertension at every HIV treatment visit and annual screening for diabetes among PLHIV on ART [33]. In contrast to Malawi and Zambia, since 2011 South Africa has implemented a “single stream” approach for all chronic conditions, including HIV and NCDs. In South African primary healthcare facilities, all chronic patients are served within the same clinic, and services for multiple chronic conditions are supposed to be provided jointly [34–36]. Further details about current national guidelines in the three study countries are presented in S1 File).

### Definitions

Two definitions are important for this analysis. First, for clarity in reporting, we used the term “clinic” to describe the specific space and time within a healthcare facility where a specified service is offered. An “HIV clinic” is thus typically the waiting area, consultation rooms, filing rooms, registration desk, etc. that serve patients seeking HIV treatment. The HIV clinic may also provide other services, either HIV-related (like testing) or for other conditions, such as TB, depending on the degree of care integration at the facility. The facility, in contrast, is the entire healthcare campus where the clinic is located. A facility typically has a number of clinics or departments, for example for maternal and child health, general outpatient care, HIV care, etc.

Second, although there are multiple definitions of “integration” used in the literature [15,19,37,38], we defined integration as the provision of non-HIV services within the HIV clinic during the same HIV treatment-related visit. This contrasts with a referral system, where a recipient of ART is referred either to another clinic within the facility, such as the outpatient department (OPD), or off site to another facility for additional NCD care. A service provided in the same facility but not in the HIV clinic is thus not considered to be integrated with HIV care, while a service that is offered within the HIV clinic, even by a separate provider or in a different room, is considered to be integrated. For example, if family planning services are offered by a dedicated family planning counselor within the HIV clinic, we would regard this as an integrated service. We considered referral from the HIV clinic to other clinics in the same facility or other facilities as a non-clinical support integration activity, even if the service clients were being referred for itself was not integrated.

### Study sites and data collection

The data presented here are drawn from two studies, SENTINEL [39] and PREFER [40], which used structured facility- and patient-level survey instruments to generate evidence on differentiated models of HIV service delivery in sub-Saharan Africa.

SENTINEL was a multi-round, multidisciplinary observational study assessing the implementation and effects of differentiated service delivery (DSD) models for HIV treatment in Malawi, South Africa, and Zambia. It comprised multiple study domains, including patient and provider surveys, provider time-and-motion observations, facility-level resource-use assessments, and, from the second round onward, surveys of patients presenting for HIV testing. Each domain engaged a distinct participant group, including ART patients, healthcare providers, clinic operations managers, and HIV testing clients. The patient survey targeted ART clients who had been on treatment for more than six months and focused on experiences with DSD models and satisfaction with ART services. Data collection involved structured interviews, direct observation of provider workflows, and extraction of routinely collected electronic medical record data to link survey responses with outcomes such as retention in care and viral suppression. Three rounds of data collection have been completed to date.

The PREFER survey was conducted in South Africa and Zambia at the same sentinel sites. It was an observational mixed-methods study exploring patient characteristics and care preferences during the first six months of ART. Data collection included quantitative surveys, discrete choice experiments, focus group discussions, EMR analysis, and measurement of ART exposure through the detection of ART metabolites. The PREFER quantitative survey elicited patient experiences of HIV care and treatment preferences in the early treatment period (first six months of ART).

In the second round of the SENTINEL study’s patient and facility-level resource utilization, and in the PREFER survey, we introduced an exploratory module designed to elicit information about integration of HIV care with other conditions and services, including hypertension, diabetes, tuberculosis, cancers, respiratory diseases, family planning, and mental health. For the purposes of this analysis, we focus on integration of hypertension and diabetes care only. Respondents to the facility-level integration module were ART clinic managers or nurses/clinical officers appointed to respond on their behalf. They were asked to describe the procedures followed for ART clients who either had a prior confirmed diagnosis of hypertension or diabetes, or who screened positive for these conditions during an ART visit and required confirmatory testing and subsequent management.

The instrument also captured how routine clinic records were collected, whether HIV and NCD records for individual patients were or could be linked, whether HIV and NCD care were provided jointly during the same clinic visit, and whether any fees were charged for NCD services. Respondents were further asked about the current level of integration and, using an open-ended question, to report any challenges or barriers to integrating services at their facility. The integration instrument included both open- and closed-ended questions and is provided in S2 File.

In addition, ART patients participating in the SENTINEL and PREFER patient surveys were interviewed using a structured patient-level questionnaire focused on their experience of integrated HIV and non-HIV care. Participants reported whether they had comorbidities such as hypertension or diabetes and, if so, how frequently their visits for clinical care and medication pickup were aligned for HIV and non-HIV conditions. Participants in the PREFER survey further reported the frequency of facility visits for non-HIV care during their first six months on ART.

The SENTINEL and PREFER study sites have been described elsewhere [39,40]. The sites were selected purposively in each country, rather than aiming for national representativeness. Facilities were chosen in consultation with national and district health authorities to capture diversity in geography, patient volume, HIV treatment DSD models, and support partners, focusing on settings with high HIV burden and use of national EMR systems. While not nationally representative, the sample was intended to reflect a range of real-world service delivery contexts within each country.

In total, we purposively selected nine public sector and three mission healthcare facilities in Malawi, 18 public sector healthcare facilities in South Africa, and 11 public sector healthcare facilities and one mission healthcare facility in Zambia. Mission facilities in Malawi and Zambia are owned and managed by religious organizations but serve the same populations as public sector facilities and follow national guidelines; some charge user fees for non-HIV services. All public facilities included in this analysis do not charge user fees for both HIV and non-HIV services.

### Data analysis

Using the responses collected from providers, we first empirically categorized the NCD services offered in HIV clinics into four categories: 1) screening and/or diagnosis; 2) monitoring and/or management; 3) treatment; and 4) non-clinical support services, including referrals (Table 1).

**Table 1:** Service categories for NCD care integrated into HIV clinics.

Service category	Components of each broad service category	
	Hypertension	Diabetes
Screening and/or diagnosis	Checking vitals (height, weight, blood pressure), and patient health history and family history	
Monitoring and/or management	Laboratory/point of care tests: Urinalysis, lipid profile, renal function, liver function, random blood sugar, full blood count, creatinine, urine microscopy tests	Laboratory/point of care tests: Glucose, hemoglobin A1c, urinalysis, urine microscopy, lipid profile, random blood sugar, fasting blood sugar, liver function, renal function, creatinine tests
Treatment	Any hypertension-related medication dispensed	Any diabetes-related medication dispensed
Non-clinical support	Nutrition assessment, referrals either to the outpatient department or offsite, information, education and counselling sessions focused on prevention, risk reduction, diet, lifestyle modification, nutrition and adherence	

An HIV clinic was classified as providing a service category if it offered at least one of the services in the category. For instance, an HIV clinic offering a random blood sugar as the only diagnostic service for diabetes was considered integrated in the ‘monitoring and/or management service category for diabetes. Another HIV clinic offering both random blood sugar tests and hemoglobin A1c tests as diabetes diagnostic and management services was also considered integrated in the ‘monitoring and/or management’ broad service category.

**Fig 1:**
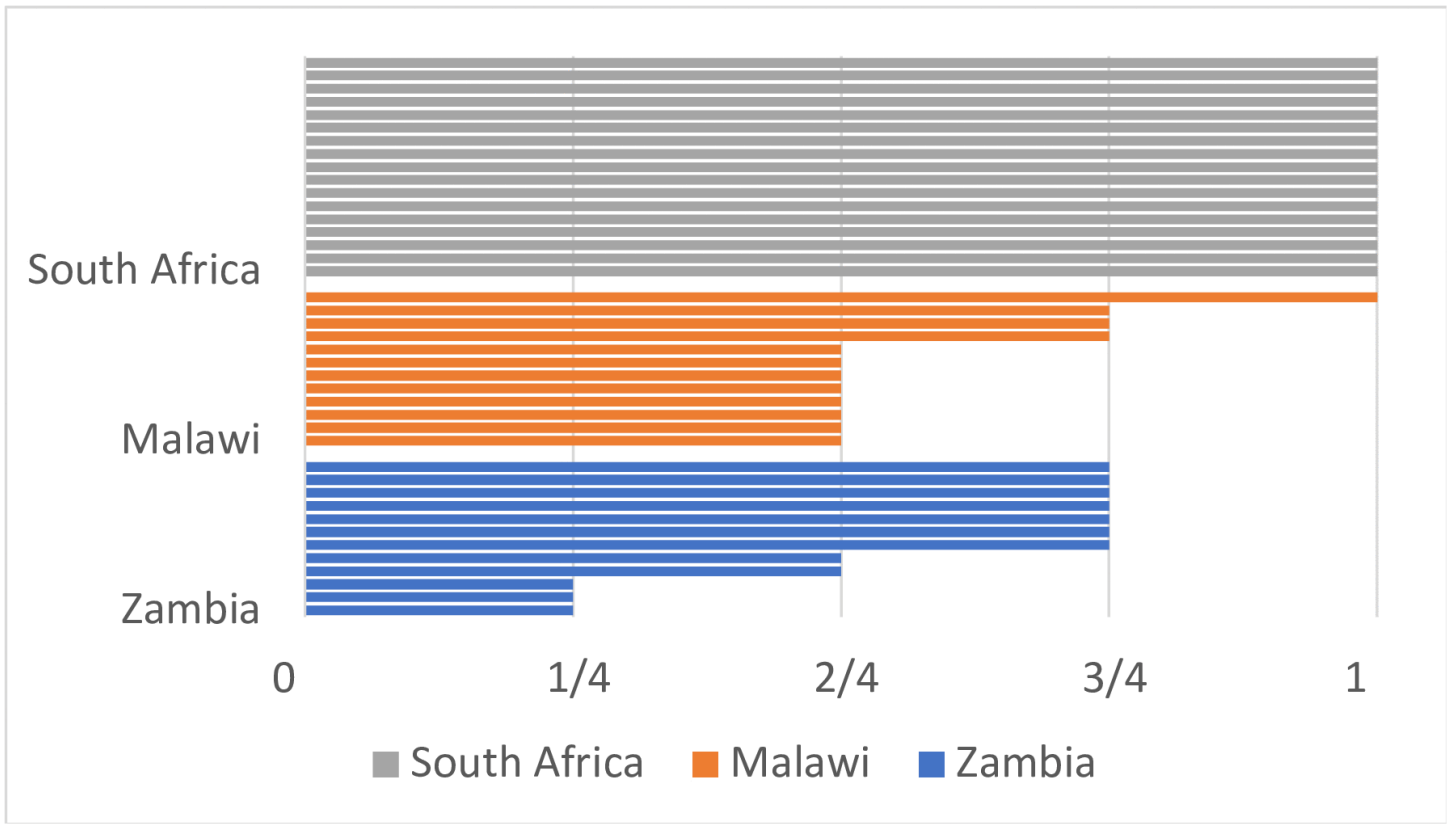
Client flow demonstrating integration completeness.

The four service categories were then used to assign a fraction of integration to each HIV clinic. We defined the integration completeness as the proportion of the service categories offered in the HIV clinic (Fig 1). If an HIV clinic did not offer any NCD services, it was defined as having “no integration” (0/4). An HIV clinic offering one (1/4), two (2/4), or three (3/4) of the service categories was defined as being “partially integrated.” An HIV clinic offering all four service categories (4/4) was defined as being “fully integrated.” For example, an HIV clinic that screened for hypertension and offered lifestyle counseling to those with high blood pressure was considered to have 2/4 (50%) integration completeness and described as being partially integrated.

Next, we assessed if facilities were more likely to integrate services for hypertension or diabetes. We observed the correlation between integration of hypertension services and integration of diabetes services at facility level using Spearman’s rank correlation coefficient. A positive and high correlation coefficient shows a positive and strong relationship between the level of integration of both conditions. A negative correlation coefficient demonstrates the likelihood of a clinic better integrating one condition over the other. We also calculated provider reported frequencies for the barriers to integration stratified by country.

Finally, we drew upon survey data collected by the SENTINEL [39] and PREFER [40] quantitative surveys of ART patients at the same study sites to summarize patient experience of care integration and alignment. Responses are reported with descriptive statistics. Data collection for both the facility-level integration and patient surveys was between 19^th^ September 2022–31^st^ July 2023.

### Ethics

Ethics approval was provided by the Boston University Institutional Review Board (Malawi H-41345, South Africa H-41402 and H-42726, Zambia H-41512 and H-42903); the University of Witwatersrand Human Research Ethics Committee (Malawi M210270, South Africa M210241 and M220440, Zambia M210342 and M210342) in South Africa; the Malawi National Health Science Research Committee (21/03/2672); and the ERES-Converge IRB (2021-Mar-012 and 2022-June-007) in Zambia.

For the component of the study where we asked for facility-level factual information only—we did not collect providers’ personal views on integration—a formal informed consent process was not required although we obtained a verbal consent from both the facility manager and the ART clinic staff responding to the instrument. Written informed consent was obtained for the individual patient surveys.

## Results

Facility-level integration data were collected from 9 public sector and three mission healthcare facilities in Malawi, 17 public sector healthcare facilities in South Africa (We were unable to collect data from one of the 18 SENTINEL facilities in South Africa due to logistical challenges hence 17 facilities.), and 11 public sector healthcare facilities and one mission healthcare facility in Zambia. The three mission healthcare facilities in Malawi charge user fees for the NCD services, while the one mission facility from Zambia does not charge user fees.

South African facilities had a median of 2,959 active ART patients (range: 1,182–7,934), compared with 3,907 (range: 2,370–13,386) in Zambia and 4,744 (range: 1,025–24,247) in Malawi. Most facilities in Malawi (58%) and South Africa (59%) were located in rural settings, whereas the majority in Zambia (67%) were urban.

### Degree of integration by facility

**Fig 2:**
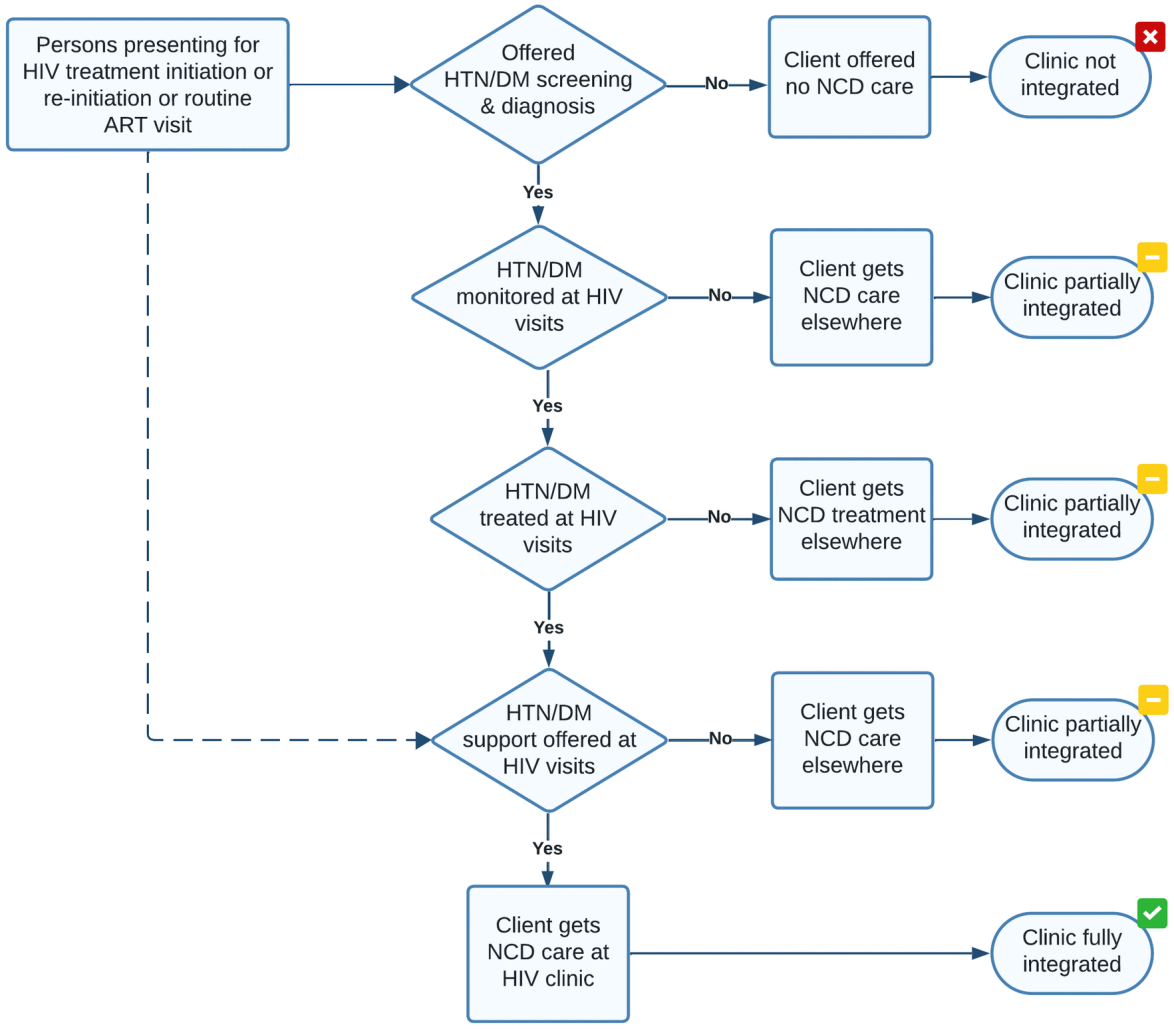
Level of integration by country. Legend: The X-axis captures each of the four broad service categories; each horizontal bar represents a single healthcare facility.

Fig 2 presents the level of integration among our sample of facilities by country. All 17 facilities in South Africa reported offering fully integrated care for both hypertension and diabetes within their HIV clinics. In Malawi and Zambia, there was wide variability in reported integration among facilities and between conditions. In Malawi, only one facility was fully integrated for hypertension and two for diabetes (the same facility was fully integrated for both hypertension and diabetes care). No facility in Zambia was fully integrated for either condition. In Malawi and Zambia, there was no noticeable difference in the degree of integration between mission and public facilities.

Table 2 further provides details about the variation in the degree of integration in Malawi and Zambia. As mentioned above, 100% of South African facilities self-reported full integration. Table 2 also indicates whether each facility is located in an urban or rural setting.

**Table 2:** NCD integration at SENTINEL facilities in Malawi and Zambia, 2023.

Integrated services provided at each study site	Setting	Hypertension				Diabetes			
		Screening and/or diagnosis	Monitoring and/or management	Treatment	Support	Screening and/or diagnosis	Monitoring and/or management	Treatment	Support
***Malawi facilities***									
M1	Rural	**✔**			**✔**				**✔**
M2^*^	Rural	**✔**			**✔**				**✔**
M3^*^	Rural	**✔**			**✔**	**✔**	**✔**		**✔**
M4	Urban	**✔**		**✔**	**✔**	**✔**	**✔**	**✔**	**✔**
M5	Urban	**✔**		**✔**	**✔**	**✔**		**✔**	**✔**
M6^*^	Rural	**✔**			**✔**	**✔**			**✔**
M7	Rural	**✔**			**✔**				**✔**
M8	Rural	**✔**			**✔**	**✔**			**✔**
M9	Urban	**✔**			**✔**	**✔**			**✔**
M10	Rural	**✔**			**✔**				**✔**
M11	Urban	**✔**	**✔**	**✔**	**✔**	**✔**	**✔**	**✔**	**✔**
M12	Urban	**✔**		**✔**	**✔**	**✔**			**✔**
**Total % integrated**		**100%**	**8%**	**33%**	**100%**	**67%**	**25%**	**25%**	**100%**
Zambia facilities									
Z1	Rural	**✔**		**✔**	**✔**	**✔**			**✔**
Z2	Urban	**✔**			**✔**	**✔**			**✔**
Z3	Urban	**✔**		**✔**	**✔**	**✔**			**✔**
Z4	Urban	**✔**		**✔**	**✔**	**✔**			**✔**
Z5	Rural	**✔**	**✔**		**✔**				**✔**
Z6	Urban	**✔**							**✔**
Z7	Urban	**✔**			**✔**	**✔**			
Z8	Urban	**✔**	**✔**		**✔**	**✔**			**✔**
Z9	Urban	**✔**							
Z10	Rural	**✔**		**✔**	**✔**	**✔**		**✔**	**✔**
Z11	Urban	**✔**							**✔**
Z12^*^	Rural	**✔**		**✔**	**✔**	**✔**		**✔**	**✔**
**Total % integrated**		**100%**	**17%**	**42%**	**75%**	**67%**	**0%**	**17%**	**83%**

* Mission facility.

For hypertension, screening and/or diagnosis within HIV clinics was universally available in both Malawi and Zambia, reported by all facilities in each country (12/12). In contrast, integrated monitoring and/or management of hypertension was uncommon, reported by only one facility in Malawi and two facilities in Zambia. Integrated hypertension treatment was more frequently available than monitoring and/or management, reported by four facilities in Malawi (33%) and five facilities in Zambia (42%). Non-clinical support services for hypertension were widely available, reported by all facilities in Malawi and by most facilities in Zambia (9/12).

For diabetes, screening and/or diagnosis within HIV clinics was less consistently available than for hypertension, reported by two-thirds of facilities in both countries (8/12). Integrated monitoring and/or management of diabetes was rare, reported by three facilities in Malawi (25%) and by no facilities in Zambia. Integrated diabetes treatment was similarly limited, reported by three facilities in Malawi (25%) and two facilities in Zambia (17%). In contrast, non-clinical support services for diabetes were widely reported, available at all facilities in Malawi and at most facilities in Zambia (10/12).

Overall, while screening and non-clinical support services for both hypertension and diabetes were widely available within HIV clinics in both countries, integrated monitoring, management and treatment services, particularly for diabetes, were offered by relatively few facilities. The extent to which integration overlapped across hypertension and diabetes is explored further in the subsequent analysis using Spearman’s correlation. Further facility-level detail is also provided in S3 File.

In all three countries, clinics that provided some degree of integrated hypertension services were also more likely to provide integrated diabetes services. The Spearman’s correlation coefficient was 0.71 for Malawi and 0.73 for Zambia (and 1.0 for South Africa). Across the eight potential services listed in Table 2, urban facilities in Malawi offered more NCD services on average than rural facilities (6.0 vs 3.7 services). This result was reversed in Zambia, where urban facilities averaged 3.4 total services and rural facilities 5.3.

### Barriers to integration reported by service providers

**Fig 3:**
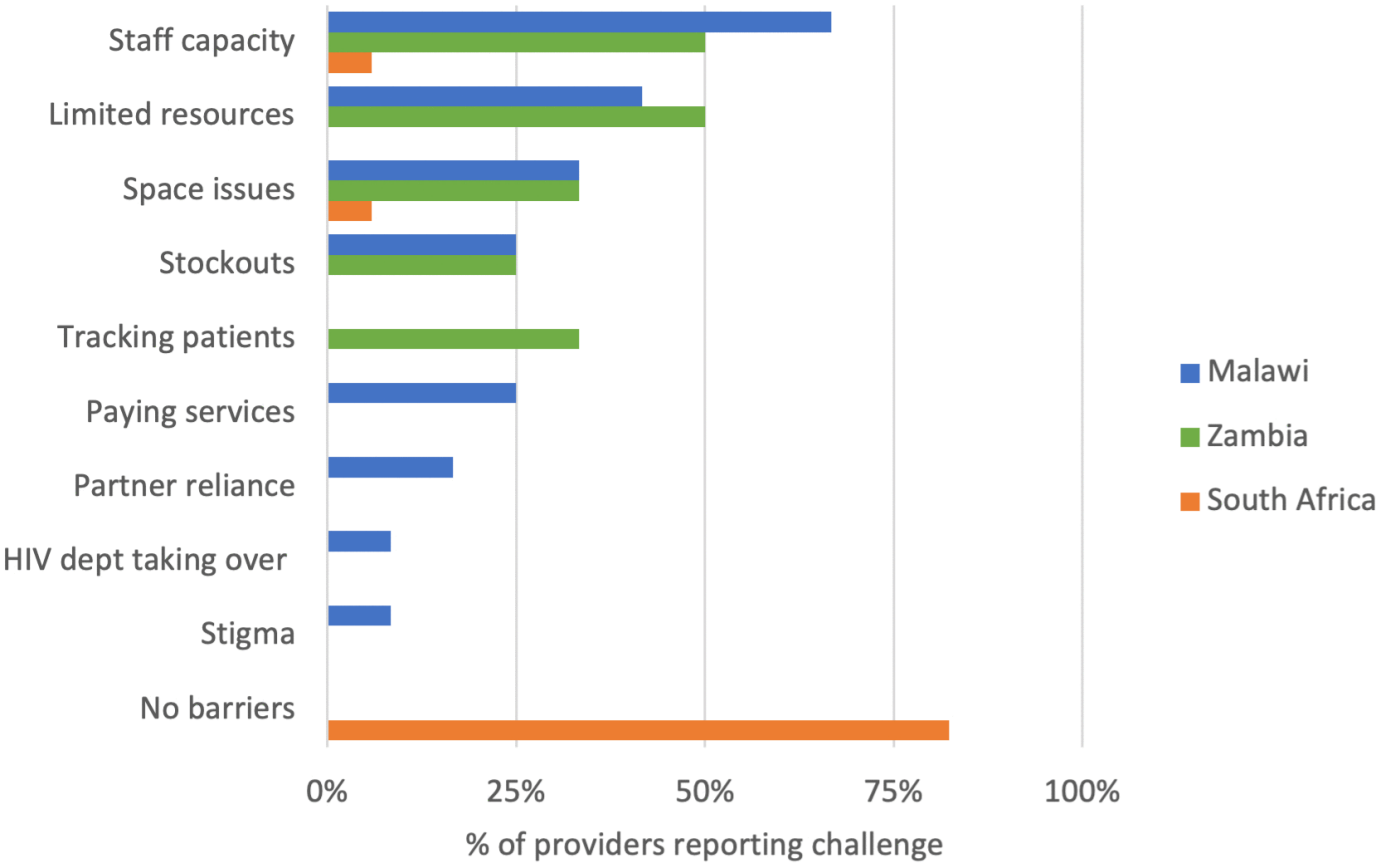
Facility-level constraints to integration as perceived by providers.

More than 80% (n = 14/17) of provider survey respondents in South Africa reported no barriers to providing integrated care (Fig 3). In contrast, more than half of the respondents in Malawi and Zambia mentioned staff capacity in terms of insufficient training and knowledge on providing integrated care and how to integrate as challenges. Other perceived barriers included space constraints in the HIV clinic and stockouts of NCD supplies. In Zambia, providers also cited difficulties in tracking patients referred from HIV clinics to the same facility’s outpatient department for NCD care as a barrier to care integration. Finally, all three mission facilities in Malawi further reported user fees for NCD services as an important barrier to providing integrated HIV-NCD care.

### Treatment clients’ experiences with integrated service delivery

Table 3 presents responses from the SENTINEL patient survey, for which 543 participants were interviewed in Malawi, 724 in South Africa, and 411 in Zambia. More than 70% of the participants in all three countries were female; median ages were 34 years in Malawi and 39 years in South Africa and Zambia.

**Table 3:** SENTINEL patient survey responses.

Variable	Malawi	South Africa	Zambia
**SENTINEL survey**			
N participating in survey	543	724	411
% female	389 (72%)	553 (76%)	288 (70%)
Median (IQR) age (years)	34 (25-45)	39 (33-47)	39 (24-48)
N (%) reporting comorbid with another chronic condition other than HIV	41 (8%)	146 (26%)	56 (14%)
% female	31 (76%)	119 (82%)	41 (73%)
Median (IQR) age (years)	37 (28-48)	47 (39-58)	46 (35-54)
**Combines HIV and chronic non-HIV visits (N=those reporting comorbid condition)**			
Often/always	19 (46%)	134 (92%)	11 (20%)
Sometimes/rarely	10 (25%)	2 (1%)	19 (34%)
Never	12 (29%)	10 (7%)	26 (46%)
**Collects HIV medication together with chronic non-HIV medication (N=those reporting comorbid condition)**			
Often/always	17 (41%)	132 (91%)	11 (20%)
Sometimes/rarely	9 (22%)	2 (1%)	13 (23%)
Never	15 (37%)	12 (8%)	32 (57%)

Among SENTINEL participants, 8% in Malawi, 26% in South Africa, and 14% in Zambia reported having another chronic condition other than HIV. Among participants with a comorbid chronic condition, more than 90% in South Africa reported being able to align HIV and non-HIV clinic visits and to combine HIV and non-HIV medication collection. In Malawi, 46% of participants with comorbid conditions reported frequently aligning HIV and non-HIV clinic visits, while 41% reported being able to align medication collection. In Zambia, 20% of participants reported alignment of both HIV and non-HIV clinic visits and medication collection.

In the PREFER survey, we interviewed 1,098 participants in South Africa and 771 in Zambia. The median age was 33 years (IQR: 27–41) in South Africa and 32 years (IQR: 27–40) in Zambia, and 72% of participants in South Africa and 67% in Zambia were female.

Overall, 8% of participants in South Africa and 12% in Zambia reported having at least one additional chronic condition alongside HIV. Because participants were in the early treatment period, HIV-related clinic visits were frequent, generally monthly in the first three months and then monthly or bimonthly thereafter. However, 70% of comorbid participants in South Africa and 44% in Zambia reported making monthly clinic visits for their non-HIV condition(s), regardless of their stage in the ART journey, suggesting relatively poor alignment of visit frequency during early HIV treatment.

## Discussion

In this descriptive analysis of the extent of integration of NCD care with HIV treatment in primary healthcare facilities in Malawi, South Africa and Zambia, we observed wide variation in the degree of integration both between and within countries. All the facilities included in our sample in South Africa reported having fully integrated NCD screening and/or diagnosis, monitoring and/or management, treatment, and support into their HIV treatment programs, and patient-reported alignment of clinical and dispensing events was very high. In contrast, no facilities in Zambia and only two in Malawi reported full integration. Integration fractions ranged from no integration at all to offering 3/4 of service categories for hypertension and/or diabetes within the HIV clinic. Patient reports from Malawi and Zambia corroborated these findings: over 50% of patients with comorbidities reported that their clinic visits and medication pickups were not aligned, and 44% in Zambia said that they had to attend the clinic every month for their non-HIV care, offsetting the benefits of multi-month dispensing of HIV medications.

Within each study country, all participating facilities operated under the same national guidelines for integrating HIV and NCD services. Despite this common policy context, we observed substantial variability in service integration across facilities within each country. In Zambia, for example, the facility fraction of integration ranged from 1/4–3/4 for both hypertension and diabetes. Although it is not surprising to find that each facility operates somewhat differently, based on its own infrastructure, human resources, patient population, and other factors, the variation between sites observed in our study calls into question the use of national averages and the accuracy of broad statements about integration at the primary healthcare level. While this study was not designed to formally evaluate implementation of national guidelines, these findings highlight the distinction between national guidelines, which outline recommended best practice, and the practical realities of service delivery within routine health system settings.

As countries in SSA continue to explore how best to deliver and scale integrated HIV and NCD care [12,14,41], South Africa’s experience with the integrated chronic disease management (ICDM) model illustrates a sustained national effort to integrate chronic disease care within primary healthcare services. Consistent with this policy environment, both providers and patients in our study reported a high degree of care alignment among established ART patients with comorbid conditions. This pattern did not extend to patients in their first six months of HIV treatment, however, when HIV and NCD clinic visits were frequent and rarely aligned. Given the high rates of disengagement from ART observed during this early treatment period [42], improved alignment of HIV and non-HIV care may represent an important opportunity to strengthen retention and outcomes for this population. Notably, fragmentation of care also appeared to persist at hospitals offering outpatient chronic disease services [16].

In contrast to South Africa, at the time of this study, Malawi and Zambia had not implemented system-wide, structural reforms to replace vertical HIV clinics with integrated chronic disease clinics. The levels of integration observed in our study therefore likely reflect both national guideline aspirations for integration and the absence of a formal national mandate for its implementation.

In both Malawi and Zambia, NCD screening and/or diagnosis and support services were more commonly delivered within HIV clinics than monitoring, management, or treatment services. This pattern is unsurprising, given the relatively low resource requirements for screening for hypertension and diabetes and for providing “support,” which often consists of lifestyle counselling, compared with the greater resources required for ongoing treatment and clinical monitoring.

In such settings, universal NCD screening in the HIV clinics, combined with an active and functional referral system for diagnosis, monitoring, and treatment, may represent the most practical first step toward integration. However, this approach carries a risk of incomplete follow-through when referrals require separate visits to other providers and are difficult for the HIV clinic to track. Challenges in tracking patients referred to other departments were also identified in this study as a key barrier to integrated care.

In addition, as more countries consider scaling up HIV–NCD integrated care, staff capacity is likely to be a key enabler [11]. In our study, more than 50% of the providers interviewed at the Malawi and Zambia sites identified limited staff capacity, particularly gaps in training and knowledge, as a barrier to offering integrated care, highlighting the importance of provider confidence and competence for effective integration. Adequate availability of other resources, including NCD screening and diagnosis supplies and laboratory tests, is also essential.

Despite the barriers to integration noted in Malawi and Zambia, providers in South Africa did not report any barriers. This finding warrants careful interpretation. While substantial operational challenges to integrated HIV and NCD care in South Africa are well documented [43,44], the low reporting of barriers in this study may reflect the framing of the survey question rather than the absence of constraints. Respondents were asked to describe barriers to integrating HIV and other chronic care services, a formulation that may have been interpreted as a process-focused question. Facilities that considered themselves to already be providing integrated care may therefore have been less likely to articulate barriers, in contrast to facilities where integration was limited or not yet implemented. As a result, the reported absence of barriers among South African facilities should be interpreted cautiously and understood as reflecting respondents’ perceptions of integration status and service organisation rather than a comprehensive assessment of operational constraints within routine care.

The lower prevalence of reported comorbid chronic conditions among participants in South Africa who were within their first six months of ART compared with those on longer-term treatment should also be interpreted with caution. This study was not designed to examine determinants of comorbidity prevalence, and we are therefore unable to distinguish between potential explanations such as differences in age, duration of engagement with the health system, or the timing of diagnosis and documentation of non-HIV conditions. As a result, this finding should not be interpreted as evidence of lower underlying need among people initiating ART, but rather as an observation that warrants further investigation in studies specifically designed to assess comorbidity detection and care over time.

Finally, despite resource considerations, integrating HIV and NCD services in LMICs appears feasible in routine care settings. Evidence from Tanzania and Uganda shows that integrated HIV and NCD service delivery did not compromise HIV treatment outcomes [15,45] and was associated with improved retention among patients with NCDs [15]. Similarly, in rural South Africa, participation in HIV treatment programmes and successful viral suppression have been associated with better awareness of hypertension diagnosis, increased NCD screening, and improved control of hypertension and blood glucose among those with diagnosed cardiometabolic disease compared with HIV-negative individuals [46,47]. This suggests that HIV treatment platforms can be leveraged to support the delivery and management of NCD services in routine care, without compromising HIV outcomes.

There are a number of limitations associated with this work. Our sample size (number of facilities included) was very small in each country and geographically restricted, making generalizability of our findings unclear despite the inclusion of both urban and rural facilities from multiple provinces and districts. Secondly, we intentionally limited our definition of integration to a “one-stop shop” model, which may not capture all approaches to integration. It is possible that some facilities do have a well-functioning referral system, such that despite certain NCD services not being offered in the HIV clinic, patients are still able to access these services elsewhere and in alignment with their HIV care.

Furthermore, the index of integration used in this study which was based on broad service categories, represents an initial and intentionally simple approach to measuring facility-level HIV and NCD integration. It captures whether services are provided within the HIV clinic but does not assess variation in the intensity, frequency, or clinical quality of care delivered within each category. As a result, clinics classified as partially integrated may differ substantially in the scope of care they provide. This measure therefore reflects structural integration rather than equivalence in the level or quality of care. Future work should build on this approach by incorporating more detailed measures of service quality and clinical outcomes.

Finally, the provider interviews were conducted with HIV clinic managers or their equivalent. Their responses may have been limited by their individual understanding of facility procedures and/or been influenced by a knowledge of what primary health clinics are supposed to offer, based on national guidelines, rather than by what their own site did offer.

## Conclusions

Despite several limitations, this exploratory study presents some of the first data available about actual integration practice for HIV and NCDs on the ground in three southern African countries. In South Africa, providers consistently reported full integration, and patients indicated a high degree of service delivery alignment. Integration varied very widely among facilities in Malawi and Zambia. In these countries, screening, diagnosis, and support services were more likely to be better integrated with HIV treatment than were monitoring, management, and treatment services. In principal, integration offers an opportunity to take advantage of the relatively robust HIV care systems [13] and successful differentiated HIV treatment service delivery (DSD) programs [14] in Malawi and Zambia to improve NCD care for the HIV-positive population. Challenges to expanding integration in the future will include insufficient staff capacity, vertically-designed infrastructure, and supply chain limitations. For Malawi and Zambia, simple alignment of clinic visits and medication dispensing, as has been successfully introduced in South Africa, may be a practical next step.

### Teaser key message

Integration of hypertension and diabetes care with HIV treatment varies widely in Malawi, South Africa, and Zambia, with greater integration in South Africa and less integration in Malawi and Zambia and little consistency across facilities.

### Key messages

- Despite standard national guidelines, the level of integration of hypertension and diabetes care with HIV treatment varies widely among primary healthcare clinics in Malawi, South Africa, and Zambia.
- While facilities in South Africa report being fully integrated, most facilities in Malawi and Zambia offer only limited screening and lifestyle support for hypertension and diabetes to clients within the HIV clinic; clients are referred out of the HIV clinic for noncommunicable (NCD) treatment and disease monitoring.
- Health program implementers encouraging integration between HIV and NCDs should consider the high level of variability between facilities at baseline so that interventions meet the facilities where they are, in order to effectively implement recommended guidelines.

## Supporting information

S1 File Summary of integration policies by country. (PDF)

S2 File Integration tool. (PDF)

S3 File Integrated services detailed by facility. (PDF)
