# Supplementary material for: Facility-level integration of hypertension and diabetes services with HIV treatment in sub-Saharan Africa: Observational evidence from Malawi, South Africa, and Zambia
Source: PLoS One. 2026 Apr 1;21(4):e0346029. doi: 10.1371/journal.pone.0346029 (PMC13042670; doi:10.1371/journal.pone.0346029)
Supplement: S1 File — (PDF) [file pone.0346029.s001.pdf]

Supplementary file 1: Summary of integration policies by country

Country	NCD policies	Diabetes policies	Hypertension policies	Source
**Malawi**	• Routinely screen ART recipients aged 40 and above for blood glucose and blood pressure<br>• HIV and NCD education integrated during group health information sessions before consultations<br>• Integrated NCD screening and management in ART services where possible, including referral<br>• Joint filing of ART and NCD patient files at the ART clinic<br>• Aligning of ART and NCD visit appointments	• Screen for diabetes and symptoms and signs of end organ damage<br>• For patient groups aged below 40, screen those with diabetes risk factors before screening using random blood glucose test<br>• For patient groups above 40, screen using random blood glucose test regardless of risk factors	• Screen all adults for hypertension<br>• Check blood pressure at least once a year for those aged 30 and above	[47]
**South Africa**	• One stop approach for all chronic conditions<br>• Integrated consultations for patients with comorbidities<br>• Integrated counselling model adapted for different conditions			[36]
**Zambia**		• Blood glucose evaluation at baseline ART visit for all PLHIV<br>• Annual blood glucose evaluation if baseline screening was normal	• Blood pressure measuring and recording at every visit	[35]

NCD, Non-communicable diseases
