# Supplementary material for: Facility-level integration of hypertension and diabetes services with HIV treatment in sub-Saharan Africa: Observational evidence from Malawi, South Africa, and Zambia
Source: PLoS One. 2026 Apr 1;21(4):e0346029. doi: 10.1371/journal.pone.0346029 (PMC13042670; doi:10.1371/journal.pone.0346029)
Supplement: S3 File — (PDF) [file pone.0346029.s003.pdf]

Supplementary file 3: Integrated services detailed by facility

Integrated services provided at each study site	Setting	Hypertension				Diabetes			
		Screening and/or diagnosis	Monitoring and/or management	Treatment	Support	Screening and/or diagnosis	Monitoring and/or management	Treatment	Support
Malawi facilities									
M1	Rural	Blood pressure check	None	None	Unspecified health education	None	None	None	Health education on diet and physical exercise
M2	Rural	Blood pressure and additional screening	None	None	Unspecified health education	None	None	None	Health education on diet
M3	Rural	Blood pressure check	None	None	Health talks on hypertension and physical exercise	Screening for diabetes	Random blood sugar	None	Health education on diet
M4	Urban	Blood pressure check	None	Drug dispensing	Counselling on diet modification	Screening for those aged over 40 and those with diabetic muscle infarction	Random blood sugar, fasting blood sugar	Drug dispensing	Health talks on diabetes prevention
M5	Urban	Blood pressure check	None	Drug dispensing	Health talks on hypertension and lifestyle modification	Screening for those aged over 40	Random blood sugar	Insulin and drug dispensing	Health education on diet
M6	Rural	Blood pressure and weight check	None	None	Unspecified counselling	Screening for diabetes and complications	None	None	Health education on diet
M7	Rural	Checking vitals	None	None	Health education on diet modification	None	None	None	Health education on diet and lifestyle modification and

Integrated services provided at each study site	Setting	Hypertension				Diabetes			
		Screening and/or diagnosis	Monitoring and/or management	Treatment	Support	Screening and/or diagnosis	Monitoring and/or management	Treatment	Support
									counselling on adherence
M8	Rural	Blood pressure check	None	None	Health education on hypertension	Screening for those aged above 40	Random blood sugar	None	Health education on diet and lifestyle modification
M9	Urban	Blood pressure check	None	None	Unspecified health education	Screening for diabetes	Random blood sugar	None	Unspecified health education
M10	Rural	Blood pressure check	None	None	Unspecified health education	None	None	None	Unspecified health education
M11	Urban	Blood pressure check	Lipid profile and urinalysis	Drug dispensing	Unspecified health education	Screening for diabetes	Random blood sugar, lipid profile, full blood count and urine dipstick	Drug dispensing	Unspecified health education
M12	Urban	Blood pressure check	Random blood sugar	Drug dispensing	Health education on hypertension and lifestyle modification	Screening for those aged above 40	Random blood sugar	None	Health education on diet, monitoring blood sugar and lifestyle modification
**South Africa**									
SA1	Urban	Blood pressure, weight, height, and pulse check	Hemoglobin A1C, urinalysis and lipid profile	Drug dispensing	Unspecified counselling	Checking vital signs	Random blood sugar, urine and hemoglobin A1c tests and lipid profile	Drug dispensing	Unspecified counselling
SA2	Rural	Blood pressure, weight, height, and pulse check	Random blood sugar, lipid profile and urinalysis	Drug dispensing	Unspecified counselling	Checking vital signs	Random blood sugar, urine glucose, hemoglobin	Drug dispensing	Unspecified counselling

Integrated services provided at each study site	Setting	Hypertension				Diabetes			
		Screening and/or diagnosis	Monitoring and/or management	Treatment	Support	Screening and/or diagnosis	Monitoring and/or management	Treatment	Support
							A1c, and lipid profile		
SA3	Rural	Blood pressure, weight, height, and pulse check	Urinalysis	Drug dispensing	Unspecified counselling	Checking vital signs	Random blood sugar, urine glucose, hemoglobin A1c, and lipid profile	Drug dispensing	Unspecified counselling
SA4	Urban	Blood pressure, weight, and pulse check	Hemoglobin A1c, and urinalysis	Drug dispensing	Unspecified counselling	Checking vital signs	Random blood sugar, and hemoglobin A1c, and lipid profile	Drug dispensing	Unspecified counselling
SA5	Urban	Blood pressure, weight, height, and pulse check	Urinalysis, thyroid, and potassium/sodium tests	Drug dispensing	Unspecified counselling	Checking vital signs	Random blood sugar, urine glucose, and hemoglobin A1c	Drug dispensing	Unspecified counselling
SA6	Rural	Blood pressure, weight and height check	Hemoglobin A1c, random blood sugar and urinalysis	Drug dispensing	Unspecified counselling	Checking vital signs	Random blood sugar, hemoglobin A1c, and lipid profile	Drug dispensing	Unspecified counselling
SA7	Rural	Blood pressure check	Lipid profile, urinalysis and creatinine test	Drug dispensing	Unspecified counselling	Checking vital signs	Random blood sugar, hemoglobin A1c, and lipid profile	Drug dispensing	Unspecified counselling
SA8	Rural	Blood pressure check	Lipid profile, urinalysis, urea and electrolyte test and full blood count	Drug dispensing	Unspecified counselling	Screening for diabetes	Random blood sugar, creatinine, hemoglobin A1c and lipid profile	Drug dispensing	Unspecified counselling

Integrated services provided at each study site	Setting	Hypertension				Diabetes			
		Screening and/or diagnosis	Monitoring and/or management	Treatment	Support	Screening and/or diagnosis	Monitoring and/or management	Treatment	Support
SA9	Rural	Blood pressure check	Lipid profile, urinalysis and routine bloods	Drug dispensing	Unspecified counselling	Checking vital signs	Diabetic foot check, random blood sugar, eye test, hemoglobin A1c test, urinalysis and lipid profile	Drug dispensing	Unspecified counselling
SA10	Rural	Blood pressure check	Lipid profile and urinalysis	Drug dispensing	Unspecified counselling	Checking vital signs	Random blood sugar hemoglobin A1c, urinalysis, lipid profile, eye test and full blood count	Drug dispensing	Unspecified counselling
SA11	Urban	Blood pressure check	Lipid profile and urinalysis	Drug dispensing	Unspecified counselling	Checking vital signs	Diabetic foot check, random blood sugar, eye test, hemoglobin A1c, and lipid profile	Drug dispensing	Unspecified counselling
SA12	Rural	Blood pressure check	Lipid profile, urinalysis and creatinine test	Drug dispensing	Unspecified counselling	Screening for diabetes	Eye test, hemoglobin A1c, random blood sugar, and lipid profile	Drug dispensing	Unspecified counselling
SA13	Urban	Blood pressure check	Lipid profile, urinalysis, and random blood sugar	Drug dispensing	Unspecified counselling	Screening for diabetes	Random blood sugar, hemoglobin A1c, lipid profile and creatinine test	Drug dispensing	Health education and diet counselling

Integrated services provided at each study site	Setting	Hypertension				Diabetes			
		Screening and/or diagnosis	Monitoring and/or management	Treatment	Support	Screening and/or diagnosis	Monitoring and/or management	Treatment	Support
SA14	Rural	Blood pressure check	Lipid profile, urinalysis, and urea and electrolytes	Drug dispensing	Unspecified counselling	Screening for diabetes	Random blood sugar, , creatinine, hemoglobin A1c,, , and lipid profile	Drug dispensing	Eye and foot care
SA15	Urban	Blood pressure check	Lipid profile, and urinalysis	Drug dispensing	Unspecified counselling	Screening for diabetes	Random blood sugar, hemoglobin A1c, lipid profile, physiotherapy, occupational therapy and foot examination	Drug dispensing	Unspecified counselling
SA16	Rural	Checking vital signs and blood pressure	Lipid profile, urinalysis, cholesterol, creatinine, and hemoglobin A1c tests	Drug dispensing	Unspecified counselling	Checking vital signs	Random blood sugar, hemoglobin A1c, and lipid profile	Drug dispensing	Unspecified counselling
SA17	Urban	Blood pressure check	Lipid profile, urinalysis, cholesterol, creatinine, and hemoglobin A1c	Drug dispensing	Unspecified counselling		Random blood sugar hemoglobin A1c, urinalysis, and lipid profile	Drug dispensing	Unspecified counselling
Zambia									
Z1	Rural	Blood pressure and clinical checkup	None	Drug dispensing	Adherence counselling	Checking for vitals	Random blood sugar and hemoglobin A1c tests	None	Nutrition and adherence counselling

Integrated services provided at each study site	Setting	Hypertension				Diabetes			
		Screening and/or diagnosis	Monitoring and/or management	Treatment	Support	Screening and/or diagnosis	Monitoring and/or management	Treatment	Support
Z2	Urban	Blood pressure check	None	None	Prevention and adherence counselling; and health education	Screening for diabetes	None	None	Dietary modification counselling
Z3	Urban	Blood pressure check	None	Drug dispensing	Nutrition assessment counselling	Screening for diabetes	None	None	Nutrition assessment
Z4	Urban	Blood pressure check	None	Drug dispensing	Information and education session	Screening for diabetes	None	None	Nutritional assessment
Z5	Rural	Blood pressure check	Liver functioning test	Drug dispensing	Referral to OPD depending on blood pressure level	None	None	None	Information, education and counselling on nutrition management
Z6	Urban	Blood pressure check	None	None	None	None	None	None	Unspecified counselling
Z7	Urban	Blood pressure check and additional screening	None	None	Unspecified counselling	Screening for diabetes	None	None	None
Z8	Urban	Blood pressure check	Urinalysis	None	Information, education and unspecified counselling	Screening for diabetes	Random blood sugar	None	Information, education and unspecified counselling

Integrated services provided at each study site	Setting	Hypertension				Diabetes			
		Screening and/or diagnosis	Monitoring and/or management	Treatment	Support	Screening and/or diagnosis	Monitoring and/or management	Treatment	Support
Z9	Urban	Blood pressure and weight check, checking family history and lifestyle	None	Drug dispensing	Health education on prevention and risk reduction and lifestyle modification	None	None	None	Health education and adherence counselling
Z10	Rural	Blood pressure and weight check	None	None	None	None	None	None	None
Z11	Urban	Blood pressure check	None	None	None	Screening for diabetes	None	Drug dispensing	Unspecified counselling
Z12	Rural	Checking of vital signs including blood pressure	None	Drug dispensing	Unspecified counselling	Screening for diabetes	None	Drug dispensing	Unspecified counselling
